# Proactive udder health management in South Africa and monitoring of antibiotic resistance of *Staphylococcus aureus* in dairy herds from 2001 to 2010

**DOI:** 10.4102/jsava.v89i0.1490

**Published:** 2018-05-09

**Authors:** Joanne Karzis, Inge-Marie Petzer, Edward F. Donkin, Vinny Naidoo

**Affiliations:** 1 Department of Production Animal Studies, University of Pretoria, South Africa; 2 Department of Animal and Wildlife Sciences, University of Pretoria, South Africa; 3 Department of Paraclinical Sciences, University of Pretoria, South Africa

## Abstract

Antibiotic resistance of strains of *Staphylococcus aureus* isolated from bovine milk is of concern internationally. The objective of this study was to investigate trends of resistance of *S. aureus* to antibiotics administered to dairy cows in 19 South African and one Zambian dairy herds (participating in the South African proactive udder health management programme) and to identify possible contributing factors. The resistance of *S. aureus* strains to eight commonly used antibiotics in South Africa from 2001 to 2010 was evaluated. *Staphylococcus aureus* isolates (*n* = 2532) were selected from cows with subclinical mastitis in 20 herds routinely sampled as part of the proactive udder health management programme. The isolates were selected from milk samples that had somatic cell counts more than 400 000 cells/mL and were tested for antibiotic resistance using a standard Kirby–Bauer test with published clinical breakpoints. The prevalence of antibiotic resistance was evaluated as a percentage of *S. aureus* isolates susceptible out of the total numbers for each antibiotic selected per year. *Staphylococcus aureus* showed a significant increase in percentage of susceptible isolates over time for all antibiotics tested except for ampicillin. The overall prevalence of mastitis did not change during the study period. However, the prevalence of mastitis caused by *S. aureus* (mostly subclinical cases) in the selected herds decreased numerically but not significantly. Reduction in the incidence of antibiotic resistance shown by *S. aureus* was presumed to be a result of the application of the proactive udder health management programme. The fact that the overall prevalence of mastitis was kept stable was possibly because of the influence of the management programme in conjunction with the return of infections caused by non-resistant strains.

## Introduction

South Africa is a developing country in southern Africa. With a population of 55 million people, the average annual milk consumption has been estimated to be approximately 36 L of milk per capita, which is well below the 200 L per capita annually recommended by the World Health Organization (Lassen 2012). Currently 98% of the country’s needs is locally produced, with approximately 10 million litres being imported (Lassen 2012). The South African milk-producing herd was estimated to be approximately 2474 dairy herds in 2012, with an average herd size of 238 and an average production of 20.2 L of milk per cow per day (Milk South Africa 2013), either using thrice or twice daily milking routines. Over the last 10 years, the number of milk producers has decreased, with an increase in average herd size (Milk South Africa 2013).

Mastitis remains the single largest contributor to losses in revenue for dairy producers worldwide (Awale et al. 2012), estimated at approximately $35 billion (Modi et al. 2012). Estimated milk production losses in a specific herd associated with elevated quarter milk somatic cell counts (SCC) of all lactating cows in the herd were estimated as an annual milk loss of 46 190 L valued at R205 544.84 (Petzer et al. 2016). In South Africa, based on investigation of routine milk samples, the prevalence of mastitis increased from 8.1% in 2002 to 15.4% in 2006 (Petzer et al. 2009). In the herds selected for this study, the overall mastitis prevalence remained stable and did not increase (Petzer et al. 2009).

The most common mastitis pathogens are found in the udder tissues and are spread between cows (contagious or host-adapted pathogens) or from the environment (environmental pathogens), such as bedding materials, manure and soil. This distinction may be important when assessing the challenges present in a specific herd as well as the measures considered to reduce or treat mastitis. Two main mastitis-causing pathogens in South Africa are *Staphylococcus aureus* and *Streptococcus agalactiae.* These organisms are termed major pathogens and are generally regarded as those commonly associated with clinical mastitis in dairy cattle. The causative pathogen should preferably be identified by laboratory testing of milk. There are other bacteria that may be present in the udder and they may have a beneficial effect by preventing the damage caused by major pathogens, because of the production of natural antibacterial substances or competition with other bacteria (Pieterse 2008). These bacteria can erroneously be implicated in instances of increased SCC and thus subclinical mastitis because they usually do not cause clinical mastitis (Schukken et al. 2003). Mastitis pathogens can infect cows both when lactating and during the dry period. Thus, it is important to identify and recognise the source of these infections in order to choose appropriate treatments. Organisms that have been identified are *S. aureus, Streptococcus agalactiae, Streptococcus dysgalactiae, Streptococcus uberis*; gram-negative major pathogens: *Escherichia coli, Klebsiella pneumonia, Serratia* spp.; minor pathogens: coagulase-negative staphylococci (non-aureus staphylococci), *Micrococcus* spp., *Staphylococcus pseudintermedius, Streptococcus pyogenes, Enterococcus faecalis, Streptococcus canis, Trueperella pyogenes* and other members of the family Enterobacteriaceae. *Staphylococcus aureus* is the principal cause of mastitis (Petzer et al. 2009, 2016).

The infected udder is considered the primary reservoir of *S. aureus* and it is believed to be transmitted during milking via contaminated teat liners, milker’s hands and communal clothes (Leslie & Schukken 1999). Once *S. aureus* infects the udder, it may cause primary clinical signs such as swelling, heat, redness, and floccules in the milk as well as abscessation and fibrosis of the udder. These bacteria may damage the secretory tissue and cause reappearance of clinical signs or elevated SCCs and may permanently limit an infected quarter’s ability to produce milk and to respond to treatment (Mellenberger & Kirk 2001). *Staphylococcus aureus* is also particularly difficult to treat effectively because it may secrete β-haemolysin, which can lead to potentially fatal gangrenous mastitis (Mellenberger & Kirk 2001). These bacteria can also avoid phagocytosis through biofilm production, which may also lead to poor antibiotic penetration (Ramadhan & Hedges 2005). The production of biofilm may also be correlated with pathogenicity and thus contribute to the virulence of individual strains (Ramadhan & Hedges 2005).

The dairy industry is a major consumer of antibiotics globally and mastitis is the most treated disease of dairy cows. In South Africa, producers have unrestricted access to 12 of 22 registered intramammary medicines without prescription, while the remaining 10 registered intramammary medicines are restricted for veterinary use (Carrington, Du Plessis & Naidoo 2016). The antibiotics available without prescription may be used incorrectly (Henton et al. 2011) and may contribute to the emergence and/or persistence of antibiotic-resistant strains in cows, humans or both (Burgos, Ellington & Varela 2005).

The proactive udder health programme of the Milk Laboratory of the Faculty of Veterinary Science, University of Pretoria, was initiated to reduce the development of resistance. The programme is comprehensive and includes routine microbiological and cytological examinations for the whole herd. This allows the early identification of *S. aureus* intramammary infections (IMI) to facilitate eradication of this organism from a herd. For this study, we report on the effectiveness of this programme on 20 herds managed consistently over a 10-year period.

## Materials and methods

A proactive udder health management programme, using the Milk Sample Diagnostic (MSD) computer programme (Abaci Systems, Aretsi SA, Pretoria), was developed and maintained over several years at the University of Pretoria (Petzer et al. 2016). The basic purpose of this management programme was to sample all lactating cows in a herd for both microbiological and cytological evaluations in order to identify *S. aureus-*positive cows. This system identified those animals which were carriers of *S. aureus* despite a low SCC. This information assisted managers and advisors in making management decisions. In this manner, cows with udder infections caused by mainly contagious bacteria could be separated from those not infected and milked last or if necessary be removed from the herd. The method implemented was thus different from other monitoring systems, where only infected quarters were sampled (Petzer et al. 2016). Producers were inducted into the programme either on their own accord or after being recruited through awareness events on the importance of proactive udder health. Participation in the programme is voluntary and paid for by the producers.

In practice, herds were sampled as frequently as possible with all cows that tested positive for *S. aureus* placed in a separate camp and milked last within 2–3 days of sampling. These cows were kept in the camp with other *S. aureus*-positive cows for the rest of their productive lives. At drying-off, cows would be removed from the herd and placed in a separate group, but returned to the group immediately after calving. The positive cows were identified differently in order to facilitate immediate identification should they be in a wrong group. They were always milked last and only after the rest of the lactating cows left the parlour, for their entire productive lives. Udders of all *S. aureus*-positive cows were palpated by an experienced veterinarian just after milking to determine possible chronic udder damage. In addition, the following criteria were used to predict the probability of cure for the individual cow: parity, stage of lactation, level of SCC in the infected quarters, numbers of quarters in an udder infected with *S. aureus* and quarter position according to a suggested formula (Sol et al. 1997). For example, there is a decreased likelihood of cure of cows in second or later lactation, within 1–99 days in milk, with high SCC (> 800 000 cells/mL), with mostly hind quarters affected and 3–4 quarters per udder affected (Sol et al. 1997). Selection of the antibiotic for treatment was based on antibiotic susceptibility testing results and cure based on culture results instead of the SCC. Microbiological cure was monitored and chronic cases were culled as soon as possible.

Milking hygiene practices included disinfection of milker’s hands or gloves that actually touched the teats (performing stripping), pre- and post-teat dipping and backwashing of clusters with effective and fast-acting disinfectant.

All milking systems were tested on an ongoing basis using both pulsographs and teat-end vacuum measurements. The following additional tests were performed weekly by managers as described by the Teat Club International (DeLaval 2017): time from touch to attachment, attachment to milk flow, maximum milk flow and percentage liner slips and residual milk. Teat-end scores were performed on first lactation cows for early detection of incorrect settings used in the maintenance of milking machines. Milking parlour staff and management were trained in implementing the practices of the proactive udder health management programme through constant communication of advice and guidance given by the laboratory manager, based on the microbiological and cytological results obtained from examinations. These dairy parlours used either the Afimilk or Alpro systems, which are highly technical systems to assist in the monitoring of parlour management.

Each herd was approached on an individual basis. Overall when the udder health status of the herd remained static or deteriorated, a veterinarian visited the herd and the protocol was immediately adjusted according to findings and observations made during the visit. This may have included re-training of milkers or managers in milking procedures, cow handling and operation; cleaning and settings of the milking machine; a new teat dip or disinfectants prescribed; the installation of a Dosatron; improvement of biosecurity or the herd kept as a closed herd thereafter; culling of specific cows; inactivation of udder quarters; introduction or continuation of vaccination with Startvac vaccine (Hipra).

This study presents the results from the first 10 years of the monitoring period. In this period, a total of 363 herds were inducted into the programme. The results from 20 of these herds (19 in South Africa and 1 in Zambia) are presented. These herds were selected because they were continually evaluated (at least 5 of the 10 years) over this period for impact of these management practices on the development of antibiotic resistance. The herd size varied from 67 to 1253 animals. All were fed on total mixed rations (TMR). The study population included mainly Holstein Friesian, Holstein crossbreeds, crossbreeds and Jersey dairy cows. Cows differed in age, parity, days in milk and milk yield.

Milk samples were taken by professional samplers or milkers trained according to the standard operating procedure (Giesecke, Du Preez & Petzer 1994). Prior to sampling, the first milk was stripped from all quarters and the teat ends were carefully cleaned and disinfected with methylated alcohol. Approximately 10 mL of milk was collected aseptically into sterile marked sample tubes and kept refrigerated until shipment. In the case of composite milk samples, the same procedure was followed, but approximately equal volumes of milk from each of the four quarters were collected in one sample tube. Samples were transported on ice to reach the Milk Laboratory within 48 h of sampling. Temperatures and conditions such as the cleanliness and appearance of sample tubes were noted on arrival at the laboratory, and samples that were spoiled or of doubtful quality were not processed. Samples were inoculated onto agar plates in the laboratory on the day of their arrival.

Initially a total of 5905 milk samples were collected (Table 1) from healthy cows and those suspected to have IMI as part of the routine testing. These samples were mainly from milk with signs of subclinical IMI (as determined by microbiological examination). Of the samples sent in for analysis, samples with vigorous growth and SCC > 400 000 cells/mL milk were selected for susceptibility testing. Intervals for whole-herd microbiological and cytological herd examinations ranged from monthly to longer intervals (Table 3).

**TABLE 1:** Total number of antibiotic susceptibility tests performed on *Staphylococcus aureus* isolates per farm, during the 10-year study period.

Herd ID	Herd size	Breed	SCC distribution at first test (ë10^3^ cells/mL milk)		Samples per year from implementation of the programme										Total per herd
			≤ 250 (%)	≥ 750 (%)	1	2	3	4	5	6	7	8	9	10	
1	83	H	64.8	2.3	1	2	-	1	2	5	2	5	5	4	27
2	294	H	46.0	49.9	1	-	4	5	5	4	4	4	3	-	30
3	239	H	52.9	25.0	7	2	6	5	5	3	1	1	-	-	30
4	699	H	43.4	14.9	1	4	3	4	3	8	7	10	11	1	52
5	296	H	45.0	10.1	1	-	1	-	1	1	2	-	-	-	6
6	329	H	42.2	19.8	2	-	4	1	4	1	3	1	-	2	18
7	624	H	80.8	7.3	1	-	1	1	-	3	-	2	-	-	8
8	300	H	79.7	10.6	2	3	1	3	-	1	-	1	2	1	14
9	233	H	50.0	40.6	1	3	2	2	1	-	3	-	-	-	12
10	1253	H	78.0	8.4	3	21	11	18	12	13	7	1	-	-	86
11	200	H	7.0	65.0	1	4	3	6	3	3	4	1	-	-	25
12	625	HX	71.0	10.7	2	1	2	-	-	-	2	7	-	-	14
13	576	H	66.6	22.7	7	1	-	1	1	3	-	7	1	-	21
14	200	HX	40.9	19.3	1	1	2	3	1	1	-	-	-	-	9
15	250	X	27.3	20.5	2	-	1	2	1	1	1	-	-	-	8
16	67	X	6.6	67.4	2	2	3	2	1	-	-	-	-	-	10
17	231	H	47.9	33.7	10	15	20	11	9	9	1	-	-	-	75
18	575	J	67.9	15.6	10	3	-	13	8	9	-	-	-	-	43
19	332	J	11.4	72.3	2	1	2	2	1	1	-	-	-	-	9
20	65	J	29.9	16.0	4	2	3	2	1	-	-	-	-	-	12

**Total per year**	**7471**	**-**	**-**	**-**	**61**	**65**	**69**	**82**	**59**	**66**	**37**	**40**	**22**	**8**	**509**

H, Holstein Friesian; HX, Holstein Friesian crossbreed; X, crossbreed; J, Jersey; SCC, somatic cell count; - , no samples tested.

Routine bacterial isolation (National Mastitis Council 2004) was performed on all milk samples in accordance with standard laboratory milk culture methodology and preliminary identification was done based on colony morphology (International Dairy Federation 1985). When fewer than two colonies were present, and no organism was identified, the milk sample was noted as ‘no growth’. When there were more than two types of organisms present, the sample was noted as contaminated (CU). Where there were two distinct groups of separate growth present, this was noted as ‘mixed growth’ (MG) or a special code was allocated when major pathogens were involved. Samples classified as ‘no growth’, CU (contamination) or mixed growth (with or without major pathogens) were not used for antibiotic sensitivity testing. The SCCs were performed by fluoro-optic-electronic methods using a Fossomatic 90 and Fossomatic 5000 (Rhine Rühr, Wendywood, Denmark). Selected isolates of pure cultures of *S. aureus* with vigorous growth from subclinical mastitis cases with an SCC of more than 400 000 cells/mL were utilised for susceptibility testing. Not all *S. aureus* isolates in the herds over the 10-year study period underwent susceptibility testing (the routine practice is to perform one susceptibility test per type of bacteria isolated per investigation).

Antibiotic susceptibility testing was performed on one *S. aureus* isolate for every herd investigation, using the Kirby–Bauer disk diffusion method (Bauer et al. 1966) for eight antibiotics with laboratory quality controls (American Type Culture Collection – ATCC No. 25923 *S. aureus*): ampicillin 10 μg (AMP), cloxacillin 5 μg (OB), penicillin G 10 IU (PEN) (beta-lactams); cephalexin 30 μg (CL), cefuroxime 30 μg (CXM) (cephalosporins); clindamycin 10 μg (DA) (lincosamides); oxytetracycline 30 μg (OT) (tetracyclines) and tylosin 30 μg (TY) (macrolides). Antibiotic susceptibility testing was performed and interpreted by measuring the zone diameter to the nearest whole millimetre for all zones of inhibition, which were categorised as susceptible, intermediate or resistant categories as clinical breakpoints established by the Clinical Laboratory and Standards Institute.

All data were initially captured in the MSD programme (Abaci Systems, Aretsi SA, Pretoria) or in Microsoft Excel. The data in the files of the MSD programme were exported into Excel as CSV files. Excel was used for data sorting and to create pivot tables and figures. All antibiotic susceptibility results that fell into the intermediate category were presumed to be resistant for the purpose of analysis of data (Wong et al. 2014). The prevalence per herd for susceptibility was listed as a percentage of all samples taken by year. As the interventions started at different times on some of the herds, this introduces a year-by-year bias into the analysis. To correct this, calculations were based on year of intervention. To ascertain if changes per year were significant among the 20 herds, binomial regression (logistic regression) was undertaken per antibiotic evaluated using the IBM SPSS statistics version 22 (IBM Corp., Armonk, NY). The chi-squared test (linear-by-linear association) was additionally conducted to verify the results obtained by binomial regression, using the IBM SPSS statistics version 22 (IBM Corp., Armonk, NY). Trends in resistance from year of programme introduction of each herd are reported.

The assumption of the model was that the longer a herd was included in the programme, the lower the prevalence of resistance of *S. aureus* to a particular drug would become.

## Results

The number of samples per herd is presented in Table 1. The first year of monitoring for a particular herd is reported as year 1 and subsequently as the years of total intervention. At 509 sampling events, a total of 815 samples positive for *S. aureus* underwent antibiotic susceptibility testing. Cefuroxime and cephalexin had the lowest percentage resistant isolates prior to any intervention on a herd at 24.32% and 15.32%, respectively. After the 10-year monitoring period, this pattern had changed with all herds, demonstrating a significant trend of decreasing incidence of antibiotic resistance (*p* > 0.05) for all products, with the exception of ampicillin, where there was no significant change (*p* = 0.104) (Tables 1 and 2).

**TABLE 2:** Data of combined resistance (%) of the *Staphylococcus aureus* isolates with 5 or more years of sampling pooled for an indication of on-farm resistance out of the total of herds sampled.

Year	AMP		CL		CXM		DA		OB		OT		P		TY	
	%	*n*	%	*n*	%	*n*	%	*n*	%	*n*	%	*n*	%	*n*	%	*n*
1	50.49	52/103	24.32	9/37	15.32	17/111	53.57	60/112	42.99	46/107	38.39	43/112	64.71	44/68	78.38	58/74
2	57.01	61/107	40.63	39/96	25.00	28/112	56.52	52/92	46.74	43/92	48.65	54/111	63.16	24/38	79.59	39/49
3	60.18	68/113	36.11	26/72	13.33	20/150	59.73	89/149	42.18	62/147	33.56	50/149	60.58	63/104	71.70	38/53
4	54.25	83/153	18.00	9/50	17.98	32/178	44.83	78/174	34.10	59/173	39.43	69/175	54.88	90/164	74.24	98/132
5	56.38	53/94	19.61	10/51	14.89	14/94	46.91	38/81	24.18	22/91	22.34	21/94	59.57	56/94	71.67	43/60
6	55.84	43/77	23.94	17/71	6.58	5/76	41.67	30/72	24.32	18/74	24.24	16/66	59.21	45/76	55.17	16/29
7	41.46	17/41	15.38	6/39	2.50	1/40	31.71	13/41	19.51	8/41	29.41	10/34	48.78	20/41	53.85	14/26
8	45.00	18/40	20.00	8/40	4.88	2/41	43.59	17/39	7.32	3/41	27.50	11/40	51.22	21/41	57.50	23/40
9	40.91	9/22	27.27	6/22	18.18	4/22	14.29	3/21	36.36	8/22	31.82	7/22	40.00	8/20	59.09	13/22
10	37.50	3/8	12.50	1/8	12.50	1/8	37.50	3/8	25.00	2/8	12.50	1/8	37.50	3/8	50.00	4/8
p*	0.104		0.004		0.005		> 0.000		> 0.000		0.001		> 0.000		> 0.000	
p**	0.104		0.003		0.004		> 0.000		> 0.000		0.001		0.016		> 0.000	

*n*, sample numbers.

AMP, ampicillin; OB, cloxacillin; CL, cephalexin; DA, clindamycin; P, penicillin G; OT, oxytetracycline; TY, tylosin; CXM, cefuroxime.

* , *p*-value binomial/logistic regression;

** , *p*-value chi-squared test (linear-by-linear association).

Cefuroxime (CXM) was the product that showed the lowest percentage resistance and the highest percentage susceptible samples over the study period.

The prevalence of IMI (SCC ≤ 250 000 + positive culture), irritation (SCC ≤ 250 000 + negative culture) and subclinical mastitis (SCC ≥ 250 000 + positive culture) in the absence of clinical signs is shown in Table 3. This was for all the *S. aureus* isolates isolated over the study period (Table 3).

**TABLE 3:** Health status and intramammary infections of the 20 herds on which management practices were altered as determined and projected by the Milk Sample Diagnostic programme used in practice (for all *Staphylococcus aureus* isolates).

Year	Healthy		IMI		Irritation		Subclinical		Grand total
	%	*n*	%	*n*	%	*n*	%	*n*	
2001	41.33	768	26.96	501	13.19	245	18.51	344	1858
2002	55.05	5117	25.19	2341	7.57	704	12.19	1133	9295
2003	51.34	14 768	20.91	6014	15.78	4538	11.97	3444	28 764
2004	46.09	13 339	27.35	7915	12.33	3570	14.23	4120	28 944
2005	37.20	18 500	29.18	14 508	13.62	6772	20.00	9945	49 725
2006	32.13	15 445	36.89	17 734	10.71	5150	20.26	9741	48 070
2007	30.39	9410	38.83	12 022	9.60	2973	21.18	6559	30 964
2008	37.28	12 589	24.57	8299	11.10	3748	27.05	9136	33 772
2009	48.69	7844	13.13	2115	16.59	2673	21.59	3479	16 111
2010	46.86	14 104	20.12	6054	10.84	3263	22.18	6675	30 096

*n*, sample numbers.

IMI, intramammary infection: somatic cell counts (SCC) ≤ 250 000 + positive culture; Irritation, SCC ≤ 250 000 + negative culture; Subclinical, SCC ≥ 250 000 + positive culture in the absence of clinical signs.

An example of how the proactive udder health management programme was implemented is presented in Table 4. For each defined monitoring period as shown in the table, the total cases of *S. aureus* were identified, together with first-time infections, repeat infections and animals cured (bacteriologically cured, which was defined as the absence of bacteria cultured for two consecutive examinations).

**TABLE 4:** Monitoring udder health results (*Staphylococcus aureus*) on one of the 20 herds over time.

Dates	STA total (*n*)	STA new (*n*)	STA repeat (*n*)	STA cured (*n*)
May-05	30	29	1	1
Jun-05	39	33	6	21
Jul-05	42	32	10	27
**Intervention**				
Feb-06	4	4	0	3
Mar-06	7	7	0	1
Nov-08	3	3	0	0
Dec-08	9	7	2	3
Jan-09	7	7	0	6
Aug-10	8	8	0	0
Sep-10	4	3	1	7
Oct-10	2	2	0	3
Nov-10	6	5	1	0

Herd size *n* = 231.

STA, *Staphylococcus aureus*; STA total (*n*), total cases of STA isolated per examination; STA new, first time infections of STA isolated from a cow; STA repeat, STA isolated more than twice from the same cow (probable chronically infected cows); STA cured, animal positive for STA on the previous test and negative for two or more consecutive examinations (bacteriological cure); intervention, application of proactive udder health management programme.

The overall prevalence of mastitis did not change significantly over this study period (Table 3), although a visual decrease (non-significant) in the incidence of *S. aureus* mastitis was shown in a few examples (Table 4).

## Discussion

The commercialisation of antibiotics in the 1940s has revolutionised medicine in many respects with many lives having been saved. However, the overuse of these once highly effective antibiotics has been accompanied by the rapid selection for resistant strains of bacteria (Davies & Davies 2010). A recent WHO health report has warned that resistance to antibiotics in general is a ‘global’ threat (World Health Organization 2014), and one that impacts both human health and the agricultural industry. The exposure of humans to antibiotic-resistant pathogens through agricultural products and contact with animals has increased from the beginning of the 21st century (Kluytmans 2010). This is noteworthy when compared to previous historic exposures that were limited to hospital nosocomial infections (Witte 1998). Of the various veterinary uses of antibiotics, our concern has been the high levels of resistance seen in mastitis-causing organisms in South Africa. The resistance of *S. aureus* to beta-lactam antibiotics in a limited study carried out in the KwaZulu-Natal province has been found to be 48% during this same monitoring period (Schmidt 2011). However, this cannot be compared with our results because of the limited data upon which the KwaZulu-Natal study was based.

In the past, most selection of antibiotics for mastitis therapy was reactive, with the predominant practice being the management and treatment of only cows with clinical mastitis. The idea when initiating the proactive herd management programme was to promote a change in herd management through other control strategies, with the aim of decreasing the need for antibiotic treatment. The programme allowed for the early specific identification of *S. aureus* as the causative organism of mostly subclinical mastitis, through a combination of the monitoring of both SCCs and bacterial culture (Table 1). The reason for considering both criteria was based on previous findings from our laboratory that have shown that 15.4% of *S. aureus* IMI might be missed when using SCC alone (Petzer et al. 2016). In this system, animals with a positive diagnosis were subjected to specific management practices such as changes in the order of their milking to reduce transmission to healthy animals, and initiation of treatment based on antibiotic susceptibility testing, with chronic cases being culled as soon as possible, to rid the herd of resistant bacteria that pose a risk of new *S. aureus* IMI.

This programme focused on procedures that would assist in decreasing the spread of this organism between animals as well as between animals and people. The process involved a combination of integrated herd and parlour hygiene, milker and manager education (and re-training), milking machine monitoring, early responsible treatment as well as constant supervision (Barkema, Schukken & Zadoks 2006). The importance of education in the value chain cannot be overlooked, as shown by Dufour et al. (2012), who found that the dairy producers who believed that they were already doing enough about mastitis (i.e. those who were not open to new management strategies) had a higher chance of acquiring new *S. aureus* infections in their herds. What was also important in the use of antibiotics was to convey the message that antibiotic treatment is only one aid in the treatment and prevention of clinical mastitis at the herd level.

Unexpectedly, we did not find a significant decrease in the overall prevalence of mastitis (mostly subclinical) (Table 3). This was possibly because of the influence of the management programme in conjunction with the return of infections caused by non-resistant strains.

However, a numerical decrease (non-significant) in mastitis caused by *S. aureus* was shown in a few of the examples from these herds (Table 4). Another South African study showed the overall trend of mastitis increasing from 8.1% in 2002 to 15.4% in 2006 from all routine samples during that time period (Petzer et al. 2009). However, the 20 herds in this study, with continuous evaluation and correct application of this management programme in practice (at 1–4-month intervals) for the 10-year study period, showed an overall stable prevalence of mastitis (Table 3). Only a significant reduction in the prevalence of antibiotic resistance in *S. aureus* was found (Table 2). This highlights the possibility of being able to farm successfully using less antibiotics for milk production. This management programme works well only when the information obtained is put into practice correctly. While the significant decrease in the resistance is an important finding (Table 2), it is not suggested that bacterial resistance can be reversed, but rather that the improved udder health management had naturally selected for less resistant/pathogenic strains of *S. aureus*. Therefore, we believe that the *S. aureus* strains that were identified in milk samples from these herds were being effectively removed from the infectious cycle (Table 4). This was done by successful and early treatment of both subclinical and clinical IMI, inactivation of quarters or culling of cows. The risk of new infections by these bacteria was also limited by isolating infected cows, by milking them last and by improving milking hygiene. This was based on the epidemiology of *S. aureus* mastitis strains, which appear to come from both other cows and humans. The rationale for this was that the chronic *S. aureus*-infected animals were repeatedly infected animals that failed to respond to treatment, thus likely representing the reservoirs of resistant pathogens. It is also plausible that good hygiene limited the spread of *S. aureus* from the farm workers to the animals. In more than eight previous *S. aureus* outbreaks in the country, a link has been shown between bacteria isolated from throat swabs of dairy parlour staff and from cows’ udders (Petzer et al. 2009).

The change in bacterial population resulting from good biosecurity measures is not an unknown phenomenon. The best examples come from intensive care units of hospitals that have implemented good hygiene practices (Sydnor & Perl 2011). In one medical study, a 9% year-on-year decrease in methicillin-resistant *S. aureus* (MRSA) cases was reported (Kallen et al. 2010). These medical studies have illustrated how the resistance profiles of bacteria can change under intensive care unit biosecurity programmes. As for the reason for the change in resistance (Table 2), this is most likely because of a replacement of the more resistant pathogens with environmental or ‘wild type’ strains of bacteria that are not yet antibiotic resistant. The term ‘wild type’ refers to the phenotype of the typical form of a species as it occurs in nature. Originally, the ‘wild type’ was conceptualised as a product of the standard ‘normal’ allele at a locus, in contrast to that produced by a non-standard, ‘mutant’ allele, typically without resistance (Merriam-Webster’s Collegiate Dictionary 1999). The environmental pathogens are more genetically diverse and able to colonise environments in which the pathogenic organisms have been removed. While we have not yet characterised the change in phenotype of the organisms over time, the resistance profiles found after the 10-year period were very similar to the results of the South African National Veterinary Surveillance and Monitoring Programme for Resistance to Antimicrobial Drugs (SANVAD) report (Van Vuuren, Picard & Greyling 2007), which indicated surprisingly low resistance of *S. aureus* among South African dairy cows to the classes of antibiotic tested. In the SANVAD results, the greatest antibiotic resistance was recorded for gentamicin, ampicillin and enrofloxacin. In spite of this fact, 12.4% of isolates were multi-resistant, with most of them being resistant to ampicillin (Crestani et al. 2016; Van Vuuren et al. 2007). Ampicillin was the only product used in this study which did not show a significant decrease in antibiotic resistance over the study period (*p* = 0.104) (Table 2).

Despite the introduction of management practices, the overall prevalence of mastitis was not significantly reduced (Table 3). Mastitis is a complex disease involving interaction between the stress experienced by the cow, the level of milk production, parity, immune competence, udder conformation and parlour management (milking machine factors and parlour hygiene). This suggests that merely controlling the external variables such as the milking machine pressure and general hygiene is not in itself sufficient to reduce the overall prevalence of mastitis significantly, although mastitis caused by *S. aureus* decreased numerically (non-significant) during the study period (Table 4) (Petzer et al. 2009). While techniques of good hygiene and management have been used for the control of mastitis in dairy herds for many years (Neave et al. 1969), this has now also been shown to have an impact on antibiotic resistance, through prudent treatment selection criteria and protocols based on susceptibility testing, which can assist dairy producers to manage effectively and eliminate *S. aureus* udder infections. These aspects should be integrated with management techniques such as general farm hygiene, milker education and supervision, routine assessment of all cows using both microbiology and cytology and strict culling programmes (Petzer et al. 2016). Attention should also be given to cow factors, and selecting for those factors that are likely predictors of resistance against mastitis. This would require that the management system should include the collection of more information related to these predictors in the management programmes. Such predictors should be determined in future studies where other associated factors in mastitis such as volume of milk produced, udder confirmation and general health could be considered.

## Conclusion

The findings of this study indicate that proactive dairy herd management for mastitis control can have a major beneficial influence on the population of bacteria. This type of information will assist in the development of government and industry policy on access and use of antibiotics and inform users on some general aspects and trends of effective antibiotics. Through this proactive udder health management practice, the results show that currently used antibiotics can still be effective. The attitudes of producers and their staff also play an important role. While it is believed that these results indicate that change in management practices is a valuable tool, these types of interventions are only of value if all role players are properly integrated into the process.
